# Supplementary material for: Exploring the Health-Related Quality of Life and the Lived Experience of Adolescents Following Invasive Meningococcal Disease
Source: Healthcare (Basel). 2024 May 24;12(11):1075. doi: 10.3390/healthcare12111075 (PMC11172222; doi:10.3390/healthcare12111075)
Supplement: Supplementary file 1 [file healthcare-12-01075-s001.zip › healthcare-2982143-supplementary.pdf]

Supplementary Materials

AMEND Semi-structured Interview Schedule

Introduction, Purpose of Interview, Confidentiality

Thank you for agreeing to be interviewed about your experience of Invasive Meningococcal Disease and its impact on your life. Please let me know if you want any questions clarified or if you wish to stop the interview at any stage. What you tell me will be completely confidential. I would like to record this interview so we can be sure we have an accurate record of this conversation. The recording will be transcribed and we will use the data for this research, however you will not be personally identified in the transcript documents, this is to protect your privacy. Are you happy for me to record this conversation? This interview should take no longer than one hour.

Questions/Prompts

General questions about yourself and family

I'd like to you tell me a bit about yourself and family. (N*umber of people in household, family members, age of participant and siblings)*

Diagnosis, treatment, time spent in hospital

Let's now talk about your experience of meningococcal disease

- When did it happen?
- Could you tell me about the circumstances of the illness?
    - Where were you when you started to feel unwell?
    - Can you describe the symptoms that resulted in you seeking medical attention?
    - What happened when you started to feel unwell (what did you and others do)?
- How did you feel emotionally when you first presented with meningococcal disease?

Hospitalisation

- How long did you require treatment for meningococcal disease?
- What type of treatment did you require? For how long?
- Can you describe what the experience was like for you and your family when you were unwell and admitted to hospital (prompt: social, emotional, and physical)

Discharge

- How did you feel when you got home?
- Can you tell me about any impairments you had after being discharged? (Prompt: How were these managed?)
- Do you feel like you got back to your pre illness level of health and functioning? (prompts: social, emotional, and physical) If so, how long did that take?

Supplementary appendix 1

Medical professionals, other organisations, family, friends, and other support sources such as social media. Burden on parent/caregiver

- Thinking back to the experience can you tell me about your thoughts on the support you received? (prompts: social, emotional, and medical)
    - Can you describe what information you were given about the condition at the time? Did you feel like it was enough? If not, what other support or information would you like to have received?
    - Have you sought out information for yourself? If so, where did you go to find it and was it any good?
- What has it been like since the illness for you and your family? (prompts: social, emotional, financial, medical issues)

Work opportunities, social aspects, education, finance, emotional and mental impact.

- Are you currently requiring ongoing treatment because of your meningococcal illness and if so, please describe
- Does your past illness impact your daily life and if so, how? (prompts: social, emotional, financial, medical issues)
- How would you say the experience has affected you personally in the longer term? Has it changed you and if so, how?
- Is there anything you would like to be able to do but because of your meningococcal illness you are unable to?
- What do other people think about you having had meningococcal disease?

Your knowledge of IMD and advice to others

- What do you know about how the disease is transmitted?
- Are you aware of any ways to reduce the risk of contracting meningococcal disease?
- What advice would you give 15 to 24 year olds about meningococcal disease?

Is there anything else you would like to tell me about or add about your experience with meningococcal disease?
